# Comparison of parental caregivers and associated factors with caregiver burden in rare paediatric diseases

**DOI:** 10.1186/s13023-026-04570-5

**Published:** 2026-10-12

**Authors:** Maja Brandt, Laura Inhestern, David Zybarth, Ramona Otto, Martin Härter, Corinna Bergelt

**Affiliations:** 1 Department of Medical Psychology, University Medical Center Hamburg-Eppendorf, Martinistraße 52, 20246 Hamburg, Germany; 2 German Center for Child and Adolescent Health, University Medical Center Hamburg-Eppendorf, Martinistraße 52, 20246 Hamburg, Germany; 3 Department of Medical Psychology, University Medicine Greifswald, Walther- Rathenau- Straße 48, 17475 Greifswald, Germany; 4 Competence Center for Epidemiology and Health Services Research for Healthcare Professionals (CVcare), Institute for Health Services Research in Dermatology and Nursing (IVDP), University Medical Center Hamburg-Eppendorf, Martinistraße 52, 20246 Hamburg, Germany

**Keywords:** Parental caregivers, Caregiver burden, Rare pediatric diseases, Psychosocial factors, Quality of life, Depressive symptoms, Care needs, Cross-sectional study

## Abstract

**Background:**

Caregiving for children with rare diseases is associated with substantial psychosocial and practical burden, yet evidence on associated factors of caregiver burden across heterogeneous rare disease populations remains limited.

**Methods:**

In this cross-sectional study, 123 parents (84% female) of children with rare diseases were recruited from specialised centres and patient advocacy organisations in Germany. Parents were descriptively analysed and compared according to the presence of disease-related care needs. Factors associated with caregiver burden among parents of children with care needs were examined using multiple linear regression.

**Results:**

Overall, 23% of parents reported borderline or clinically relevant depressive symptoms and 77% reported at least one unmet supportive care need. Within the care needs subgroup, 24% reported high caregiver burden. After adjustment for multiple testing, group differences between parents of children with and without care needs were not statistically significant for depressive symptoms, unmet supportive care needs, or treatment satisfaction, although medium effect sizes indicated potentially meaningful differences. Children with care needs were significantly younger, had earlier symptom onset, and tended to show higher symptom severity. Lower care-related quality of life, higher socioeconomic status, parental depressive symptoms, and lower treatment satisfaction were identified as the strongest factors associated with caregiver burden.

**Conclusions:**

Caregiver burden in parents of children with rare diseases in Germany is multifactorial and shaped by both child- and parent-related factors. The unexpected association between higher socioeconomic status and greater caregiver burden requires further investigation. These findings support the need for systematic caregiver burden screening and holistic, family-centred interventions across all socioeconomic groups.

## Background

Rare diseases affect an estimated 30 million people across Europe, with the majority of cases manifesting during childhood [1]. These conditions are often chronic, progressive, and associated with significant physical, emotional, and social challenges—not only for the affected individuals but also for their families [2]. Parents of children with rare diseases frequently take on the role of primary caregivers, navigating complex medical systems while managing the day-to-day demands of their child’s care. This caregiving role is often accompanied by substantial emotional strain, financial pressure, and social isolation [3–5].

Compared to parental caregivers of children with more common chronic conditions, parents of children with rare diseases often face additional challenges, including delayed diagnoses, limited treatment options, a lack of disease-specific information, and inadequate professional support [6, 7]. These stressors may contribute to elevated levels of psychological distress and caregiver burden [2, 8].

Several studies have investigated the burden on caregivers in certain rare diseases or identified factors that contribute to a reduced quality of life due to the caregiving situation [8, 9]. More general framework models of caregiver burden, such as the Conceptual Model of Caregiving Process and Caregiver Burden Among Pediatric Population by Raina et al. [10] emphasize that caregiving is a dynamic, multidimensional process involving a complex interplay of factors that influence caregiver burden. The central idea of Raina and colleagues’ model is that caregiver outcomes are not solely determined by the severity of the child’s condition but are also significantly shaped by the caregiver’s personal resources and the availability of support systems. However, empirical research applying more conceptual framework models to the situation of parental caregivers of children with rare diseases remains scarce.

Understanding the psychosocial situation of parental caregivers is essential for developing targeted support strategies and healthcare interventions. Identifying the factors that contribute to caregiver burden in particular, may help healthcare providers and policymakers in designing services that are more responsive to the unique needs of families affected by rare diseases [11, 12]. Therefore, the aim of the present study is to gain a deeper understanding of the situation of parental caregivers of children with rare diseases. By comparing parental caregivers of children with a rare condition with and without care needs, we seek to explore differences, thereby contributing to a more nuanced understanding of the specific burdens faced by these families. Furthermore, within the group of parental caregivers whose children require care due to their rare condition, we analyse which factors are most strongly associated with caregiver burden.

## Methods

This study employed a cross-sectional comparative design to examine differences in various outcome variables (see below) between parental caregivers of children with and without care needs. Both groups included parents of children with rare diseases and differed according to the presence or absence of disease-related care needs as reported by the parents. Within the subgroup of parents of children with care needs, multiple linear regression analyses were further conducted to explore factors associated with caregiver burden.

We conducted an exploratory secondary analysis based on data collected from parental caregivers of children with rare diseases. The data were originally collected as part of the project *“Evaluation of interface management concepts for rare diseases: systematic assessment of interface management processes and development of best-practice recommendations (original title: Evaluation von Schnittstellenmanagementkonzepten bei Seltenen Erkrankungen: Systematische Bestandsaufnahme & Erstellung von Best-Practice-Empfehlungen)”.* The design and methodology of the original study have been described in detail in the study protocol by Inhestern et al. [13]. The underlying research project employed a mixed methods design to explore and evaluate the experiences and perspectives of patients, parental caregivers of paediatric patients and key stakeholders regarding intersectoral collaboration and communication in the care of individuals with rare diseases.

### Data collection and participants

Eligible participants in the original study were patients or parents of paediatric patients diagnosed with, or suspected to have, a rare disease or adult patients affected by a (suspected) rare disease. Participants with a suspected rare disease were included if they were already receiving care in a specialized rare disease centre or outpatient clinic, as diagnostic processes in rare diseases are often prolonged and, in some cases, a definitive diagnosis cannot be established despite extensive diagnostic evaluation and substantial disease-related burden [14].

Participants were recruited via specialized centres for rare diseases or a rare disease outpatient clinic in Germany. These centres or clinics were pre-selected based on a positive pre-evaluation of their intersectoral management concepts [13]. Eligible participants were invited for study participation. Those individuals who expressed interest in participation received detailed study information, a paper-based questionnaire, as well as a pre-paid, self-addressed return envelope to send the completed survey documents to the study team. Further, patient organizations distributed study information to their members, and interested individuals contacted the study team directly to receive the study documents by mail. Data collection was conducted over a 12-month period, from January 2021 to January 2022.

The original study by Inhestern et al. [13] employed a mixed-methods design and included multiple participant groups. The quantitative data collection within the original study project comprised adult patients with a confirmed or suspected rare disease (*n* = 176) and parents of paediatric patients with a confirmed or suspected rare disease (*n* = 123).

For the present work, a secondary analysis was conducted using only the subsample of parental caregivers of children with rare diseases (*n* = 123). Adult patient data and all other participant groups from the original study were excluded from the present analyses. The sample predominantly consisted of mothers and represented a predominantly medium- to high-socioeconomic-status group. Detailed demographic, socioeconomic, and clinical characteristics are presented in the Results section and Tables 1a and 1b.

**Table 1a:** Demographic and characteristics among 123 parents of children suffering from a rare disease with and without care needs in Germany

**Characteristic**	**Total**		**Without care needs**		**With care needs**		**Statistical test**		
	*N*	%	*n*	%	*n*	%	Test value	*p*	*p FDR*
**Parents**									
Total	123	100	61	49.6	62	50.4			
Gender: female	103	83.7	53	86.9	50	80.6	*χ*^2^ = .879	.384	
Mean age (SD)		39.8 (7.1)		41.1 (7.6)		38.5 (6.4)	*t* = 1.82 (*df* = 100)	.071	.142
*Family status*:									
Single Married/ living in partnership Divorced/ separated Widowed	19 90 8 3	15.8 75.0 6.7 2.5	13 41 3 2	22.0 69.5 5.1 3.4	6 49 5 1	9.8 80.3 8.2 1.6	LR = 4.165 (*df* = 3)	.244	
*School leaving qualification*:									
Secondary school ^a^ High school ^b^ Other	39 75 16	33.0 63.5 3.3	17 38 3	29.3 65.5 5.1	22 37 1	36.7 62.7 1.7	LR = 7.721 (*df* = 5)	.172	
*Occupational status*									
Full-time Part-time Homemaker Occupational disability pension Old-age pension/ early retirement Other ^c^	29 67 7 2 1 17	23.6 54.5 5.7 1.6 0.8 10.6	13 39 3 2 - 6	21.3 63.9 4.9 3.3 - 9.9	16 28 4 2 1 11	25.8 45.2 6.5 3.2 1.6 17.7	LR = 10.020 (*df* = 7)	.187	
*Monthly income*									
Under 1250 1250 – < 1750 1750 – < 2250 2250 – < 3000 3000 – < 4000 4000 – < 5000 >/= 5000	2 2 9 11 28 35 32	1.7 1.7 7.4 9.1 23.1 28.9 26.4	1 - 6 6 15 15 16	1.7 - 10.0 10.0 25.0 25.0 26.7	1 2 3 5 13 20 16	1.6 3.3 4.9 8.2 21.3 32.8 26.2	LR = 7.507 (*df* = 8)	.483	
Winkler’s Social Status Index		15.0 (3.8)		15.0 (3.9)		15.0 (3.8)	*t* = − .047 (*df* = 120)	.962	.962
*Winkler’s social status*									
Lower class Middle class Upper class	5 50 67	4.1 41.0 54.9							
Affected by a severe physical illness	16	13.0	8	13.1	8	12.9	*χ*^2^ = .001	.971	
Received psychotherapeutic or psychological support	49	40.2	20	32.8	29	47.5	*χ*^2^ = 2.763	.096	
Number of underaged children living in household (SD)		1.75 (.7)		1.75 (.8)		1.74 (.7)	*t* = .123 (*df* = 120)	.902	1.000
HADS–Depression (SD)		4.9 (4.0)		4.1 (3.5)		5.8 (4.3)	*t* = -2.838 (*df* = 121)	**.019 (*d*** ** = − .43)**	.076
*Depressive symptoms (HADS-D)*									
Unremarkable (0–7) Borderline (8–10) Clinically relevant (≥ 11)	94 15 14	76.4 12.2 11.3							
WHO-QOL-Bref–Physical health (SD)		76.1 (15.8)		72.2 (16.0)		70.1 (18.4)	*t* = .669 (*df* = 120)	.365	.584
OSSS-3–Social support (SD)		10.3 (2.3)		10.2 (2.2)		10.4 (2.5)	*t* = − .477 (*df* = 120)	.634	.846
ZUF8–Treatment satisfaction (SD)		29.3 (3.2)		30.0 (2.6)		28.6 (3.6)	*t* = 2.483 (*df* = 115)	**.014 (*d*** ** = .46)**	.112
SCNS-ST9–Unmet care needs (SD)		32.0 (25.9)		27.2 (23.4)		36.8 (27.5)	*t* = -1.983 (*df* = 110)	**.050 (*d*** ** = − .38)**	.133
*SCNS-ST9–Number of unmet care needs*									
0 1–2 3–4 ≥ 5	26 18 26 42	23.2 16.1 23.2 37.5							
*SCNS-ST9–Domain-specific unmet needs (moderate/high*,* ≥ 4)* ^d^									
Physical/daily living Psychological Support Information Sexuality	48 46 39 37 16	39.0 37.4 31.7 30.1 13.0							
ZBI–Caregiver burden (SD) ^e^						14.5 (8.8)			
*ZBI categories* ^e^ No to mild Mild to moderate High burden					22 25 15	35.5 40.3 24.2			
CarerQoL–Care-related quality of life (SD) ^e^						9.4 (2.8)			

Note. *p FDR*: p-values for t-tests were adjusted using the Benjamini–Hochberg False Discovery Rate (FDR). *LR*: Likelihood ratio was calculated in the case that the cross-table cells had an expected frequency of < 5. HADS–D: Hospital Anxiety and Depression Scale–Depression subscale; ZAPA: Questionnaire assessing satisfaction with outpatient care with a focus on patient participation; SCNS-ST9: Screening Tool for Supportive Care Needs; OSSS-3: Oslo Social Support Scale

^a^ Secondary school or junior high school

^b^ Technical college qualification or A-levels

^c^ Currently unemployed, housewife or -husband, on parental leave, in training/retraining or another status

^d^ Moderate-to-high unmet needs were defined as SCNS-ST9 item scores ≥ 4

^e^ Assessed only for parents of children with care needs

**Table 1b:** Demographic and characteristics among 123 children suffering from a rare disease with and without care needs in Germany

Characteristic	Total		Without care needs		With care needs		Statistical test		
	*N*	%	*n*	%	*n*	%	Test value	*p*	*p FDR*
**Children with RD**									
Total	123	100	61	49.6	62	50.4			
Gender: male	71	59.2	31	25.8	40	33.3	*χ*^2^ = 2.108	.147	
Mean age (SD)		6.9 (5.6)		8.0 (6.1)		5.8 (4.9)	*t* = 2.447 (*df* = 120)	**.026 ** **(*d*** **= .41)**	**.039**
Mean age of symptom onset in years (SD)		4.3 (4.5)		5.6 (5.1)		2.4 (2.8)	*t* = 2.440 (*df* = 44)	**.019 ** **(*d*** **= .73)**	**.039**
Time to diagnosis in years (SD)		4.5 (4.2)		4.9 (4.6)		4.1 (3.9)	*t* = .856 (*df* = 86)	.394	.394
*Symptom severity*									
Low Medium High Varying	23 27 53 10	20.4 23.9 46.9 8.8	15 15 18 5	13.3 13.3 15.9 4.4	8 12 35 5	7.1 10.6 31.0 4.4	LR = 7.615 (*df* = 3)	.055	
*Diagnosis* ^f^									
Marfan Syndrome VACTERL association Cystic fibrosis Oesophageal Atresia Other	20 4 11 34 21	22.2 4.4 12.2 37.8 23.3	13 - 5 17 12	14.4 - 5.6 18.9 13.3	7 4 6 17 9	7.8 4.4 6.7 18.9 10.0	LR = 7.850 (*df* = 4)	.097	
*Symptom cluster* ^g^									
Feeding/ gastrointestinal	57	46.3							
Respiratory/ airway	34	27.6							
Musculoskeletal/ connective tissue	28	22.8							
Neurodevelopmental/ cognitive/ behavioral	24	19.5							
Cardiovascular/ ophthalmologic	14	11.4							
Multiorgan/ congenital/ systemic	16	13.0							

Note. *p FDR*: p-values for t-tests were adjusted using the Benjamini–Hochberg False Discovery Rate (FDR). *LR*: Likelihood ratio was calculated in the case that the cross-table cells had an expected frequency of < 5

^f^ Confirmed diagnoses only

^g^ Multiple symptom clusters per child were possible. Symptom clusters were derived from parent-reported free-text descriptions of disease-related complaints and grouped into feeding/gastrointestinal (e.g., dysphagia, reflux, feeding difficulties, esophageal atresia-related symptoms, and growth problems), respiratory (e.g., tracheomalacia, respiratory infections, and breathing difficulties), musculoskeletal/connective tissue (e.g., scoliosis, connective tissue abnormalities, and joint instability), neurodevelopmental/behavioural (e.g., developmental delay, speech, cognitive, or behavioural problems), and multisystem domains (e.g., cardiovascular, metabolic, hepatic, or multi-organ manifestations)

### Instruments

The following instruments were completed by parental caregivers using standardized self-report questionnaires:

**Sociodemographic and disease-related variables** for parental caregivers included gender, age, income, education, and employment; for the affected child, age, gender, diagnosis, the two strongest symptoms, symptom severity (assessed using a single-item parent-report scale ranging from 0 [“low”] to 5 [“high”], while a separate response category [6] indicated varying symptom severity), and age at symptom onset. Symptom severity was recoded for further analyses: values ranging from 0 to 5 were included as an ordinal variable, while high variability in symptoms was dummy coded and included as a nominal variable in the analyses. In addition, an exploratory descriptive analysis was conducted in which parent-reported free-text descriptions of the child’s two most prominent disease-related symptoms were grouped into broad symptom clusters (feeding/gastrointestinal, respiratory, musculoskeletal/connective tissue, neurodevelopmental/sensory/behavioural, cardiovascular/ophthalmologic, and multiorgan/congenital) to further characterize the clinical presentation of the sample. Socio-economic status was measured using the Winkler-Stolzenberg-Index based on educational status, occupational status and household income [15]. The index ranges from 3 to 21 with low status (score 3–8), middle (score 9–15), and high status (score 15–21).

**Depressive symptoms** were assessed using the Hospital Anxiety and Depression Scale–Depression subscale (HADS-D), a widely used 7-item instrument for screening depressive symptoms in somatic and caregiver populations [16]. Participants answered all items on a 4-point-Likert scale, leading to possible overall scores from 0 to 21 for each of the subscale. Recommended cut-off values classify scores as unremarkable (0–7), borderline (8–10), or indicative of clinically relevant depressive symptomatology (≥ 11).

**Psychological or psychotherapeutic service utilization** was assessed via a self-report item: *“Have you ever received psychotherapeutic or psychological treatment?”* Participants who responded affirmatively were presented with a follow-up question to indicate whether the treatment was completed or still ongoing.

**Physical health-related quality of life** was measured using the Physical Health subscale of the WHOQOL-BREF, a standardized 26-item instrument developed by the World Health Organization to assess subjective quality of life across four domains: physical health, psychological health, social relationships, and environment [17]. The Physical Health subscale consists of 7 items covering key aspects such as energy and fatigue, pain and discomfort, sleep and rest, mobility, activities of daily living, dependence on medical substances and aids, and work capacity. Each item is rated on a 5-point Likert scale, with higher scores indicating better perceived physical health. Domain scores were calculated according to the WHOQOL-BREF scoring guidelines and transformed to a 0–100 scale for comparability.

Additionally, the presence of **physical illnesses** was assessed using a single item: *“Are you currently suffering from any serious physical illnesses?”*

**Perceived social support** was assessed using the German version of the Oslo Social Support Scale (OSSS-3) on the extent of perceived social support [18]. The total score of this brief instrument (3 items) ranges from 3 to 14, with higher scores indicating stronger perceived social support. Based on established cut-off values, scores can be categorized into low (3–8), moderate (9–11), and high (12–14) levels of social support.

To investigate **treatment satisfaction**, we used the ZUF-8, the German version of the Client Satisfaction Questionnaire (CSQ-8), which evaluates satisfaction with received healthcare services [19]. The instrument consists of 8 items; each rated on a 4-point Likert scale resulting in a total score between 8 and 32. Higher scores reflect greater satisfaction with healthcare services. Study participants were instructed to focus on the specialized health care of their child’s rare disease when answering the questions.

**Unmet supportive care needs** were assessed using the 9-item Supportive Care Needs Survey Short Form (SCNS-SF9/ SCNS-ST9), adapted for family caregivers, which captures key areas of insufficient support in the caregiving context [20]. The instrument includes 9 items covering multiple domains such as information needs, emotional support, healthcare access, practical assistance, and communication with healthcare providers. Respondents rate each item on a 5-point Likert scale, with higher scores indicating greater unmet supportive care needs.

To differentiate between families of children with rare diseases **with and without care needs**, parental caregivers were asked whether their child required special support in daily life as a result of their condition. An affirmative response directed parents to complete an additional section of the questionnaire addressing additional measures (see below). This approach enabled the classification of participants based on the presence or absence of caregiving demands associated with the child’s rare disease.

Additional measures were included for parental caregivers of children with care needs:

**Caregiver burden** was measured using the Zarit Burden Interview short version (ZBI-12), a validated 12-item scale that captures the subjective burden experienced by informal caregivers [21]. Each item assesses aspects of emotional, physical, social, and financial strain associated with caregiving responsibilities. Responses are given on a 5-point Likert scale, yielding a total score between 0 and 48, with higher scores indicating higher perceived burden.

**Care-related quality of life** was assessed using the CarerQoL-7D, a multidimensional tool for measuring the impact of caregiving on quality of life, developed specifically for informal caregivers [22]. The tool includes seven dimensions, covering both burdens and positive aspects of caregiving.

All instruments were used in their validated German versions.

### Analyses

Data were analysed using IBM SPSS Statistics (Version 29). In an initial step, we characterized the sample through descriptive analyses of two parental caregiver groups: those whose child had specific care needs, and those whose child had a rare disease but did not require specific care, before proceeding to between-group comparisons. Care needs were defined via parental self-report as the necessity for regular assistance or support due to the child’s condition. The comparison included all aforementioned variables, with the exception of the additional measures, which were administered exclusively to parental caregivers of children with specific care needs (see above). To assess differences between the two groups, we applied descriptive analyses and analysed group differences using t-tests for continuous data. Effect sizes (Cohen’s *d* or ф) were calculated when a significant p-value was observed. To control for multiple comparisons, p-values for t-tests were further adjusted using the Benjamini–Hochberg false discovery rate (*FDR*), which is recommended for correlated clinical and psychosocial outcomes [23, 24]. For categorical data, chi-square (χ^2^) tests were conducted. If expected cell frequencies were below 5, likelihood ratio tests were used as an alternative. Frequencies for categorical variables were reported per group only when used for statistical comparison; otherwise, categories are presented for descriptive purposes in the total sample.

In a second step, we investigated associated factors of caregiver burden within the subgroup of parental caregivers whose child had a specific care need. The selection of associated factors followed a structured three-phase approach: (1) Pre-selection of variables was guided by previous research on caregiver burden, particularly the conceptual framework by Raina et al. [10], which outlines relevant domains influencing caregiver outcomes. (2) We then examined the selected variables for their bivariate associations with caregiver burden (e.g., via correlation analyses and scatterplots) and assessed whether they met the assumptions required for linear multiple regression (e.g., multicollinearity, normality of residuals, and linearity). (3) Based on these criteria and the available sample size, six potential predictors were initially included in the first regression model. Following model diagnostics and evaluation of incremental explanatory value, predictors with negligible unique contributions were removed stepwise (first unmet supportive care needs, then physical health), resulting in a final reduced model including the four predictors with meaningful explanatory value. For all models, there was no indication of multicollinearity among predictors (all variance inflation factors [VIFs] < 1.8), and diagnostic checks confirmed that assumptions of linearity, normality of residuals, homoscedasticity, and independence were met, as recommended by Field [25]. To prevent cumulation of Type I error, the significance of independent variables was only assessed when all variables were included into the models. Semi-partial squared correlations (*sr²*) were calculated to quantify the proportion of variance in caregiver burden uniquely explained by each predictor while controlling for all others. Because the sum of *sr²* can exceed the total overall variance explained by the model (*R²*) due to correlations among predictors, the difference was defined as shared variance to ensure that the total explained variance equalled 100%. Missing data were handled using regression-based imputation, a method shown to be robust under the assumption of data missing at random (MAR) [26].

## Results

### Sample characteristics

A total of 123 parental caregivers participated in the study, including 62 parents of children with care needs and 61 parents of children without care needs. Detailed demographic characteristics and group comparisons are presented in Table 1a.

The majority of participants were female (84%), with a mean age of 40 years. Most parental caregivers were married or in a committed partnership (75%) and 55% were employed part-time and 25% full-time. Based on Winkler’s Index of Social Status, 41% of the parents were identified as belonging to the middle class, whereas 55% were identified as belonging to the upper class, suggesting that the sample predominantly represented a medium to high socioeconomic status group.

Among the children in the sample, 60% were male, with a mean age of 7 years. 21% had a suspected diagnosis. Among those with confirmed diagnoses, the most common conditions were oesophageal atresia (38%), Marfan syndrome (22%), and VACTERL association (4%). On average, symptom onset occurred at 4 years of age, and the diagnostic pathway (time from symptom onset to diagnosis) took approximately 4 years, although both measures exhibited considerable variability. Most parental caregivers rated their child’s symptom severity as severe (47%), followed by moderate (24%) and mild (20%) symptoms. Exploratory categorization of parent-reported disease-related complaints showed that feeding/gastrointestinal, respiratory, musculoskeletal/connective tissue, and neurodevelopmental/sensory symptom clusters were most frequently reported, reflecting the multisystem complexity of the conditions in this sample. Cardiovascular/ophthalmologic and multiorgan/congenital manifestations were less frequent but present. Further details are provided in Table 1a, b.

There were no statistically significant differences between the two groups in terms of demographic variables of the parental caregivers. Children with care needs were younger on average than those without care needs (5.8 vs. 8.0 years; *p_FDR =* .039, *d =* .73), and symptom onset appeared at a significantly earlier age (2.4 vs. 5.6 years; *p_FDR =* .039, *d =* .73). Children with care needs also exhibited greater symptom severity on average than those without care needs (31% vs. 16% with high symptom severity); however, this difference approached but did not reach statistical significance *(p* = .055).

### Depressive symptoms and physical health outcomes

Among all parental caregivers, 12% exhibited depressive symptoms at a borderline clinically relevant level, while 11% reported clinically significant depressive symptoms. Additionally, 40% of parental caregivers indicated that they had sought, or were currently seeking, psychological or psychotherapeutic support.

Regarding physical health, 13% of parental caregivers reported having a serious physical illness. The average score on the WHOQOL-BREF Physical Health Scale was *M* = 76.1 (*SD* = 15.8), reflecting a generally high level of perceived physical health-related quality of life among participants. Unadjusted analyses indicated that parental caregivers of children with care needs showed significantly higher rates of borderline or clinically relevant depression symptoms (*p =* .019, *d = −* .43). However, after controlling for multiple comparisons using the false discovery rate (FDR), these effects did not remain statistically significant (*p_FDR* = .076). The medium effect size (*d* = − .43) nonetheless suggests a potentially meaningful group difference despite the lack of statistical significance after adjustment. No significant group differences were observed for physical health (*p_FDR* ≥ .13). For details, see Table 1a.

### Social support, satisfaction with treatment, unmet needs and caregiver burden

Parental caregivers generally reported moderate to high levels of social support, with 51% indicating moderate support and 30% reporting high support. No significant group differences were found in social support between parents of children with versus without care needs. Regarding satisfaction with their child’s rare disease treatment, the average satisfaction score was 29.3, reflecting a high overall satisfaction. Unadjusted analyses indicated that parents of children with care needs reported significantly lower treatment satisfaction than parents of children without care needs (*p* = .014, *d* = .46). After FDR correction, this difference was no longer statistically significant (*p_FDR* = .112), though the medium effect size suggests a potentially meaningful difference.

For unmet care needs, 76.8% of participants reported at least one unmet supportive care need, and 37.5% reporting five or more unmet needs, indicating a high prevalence of multiple concurrent needs [20]. Regarding the type of unmet needs, moderate-to-high unmet needs (SCNS-ST9 item score ≥ 4) were most frequently reported in the physical/daily living (39.0%) and psychological domains (37.4%), followed by support (31.7%) and information-related needs (30.1%), while sexuality-related needs were least frequently endorsed (13.0%). In the group comparison, parents of children without care needs reported low to moderate unmet needs (*M* = 27.2, *SD* = 23.4), whereas parents of children with care needs reported moderate unmet needs in multiple domains (*M* = 36.8, *SD* = 27.5). The difference between groups corresponded to a medium effect size (*p* = .050, *d* = .38). Although this difference was not statistically significant after FDR correction (*p_FDR* = .133), it suggests a meaningful trend, highlighting that caregiving for children with care needs is associated with notably higher unmet supportive care needs. No other significant group differences were observed across these psychosocial outcomes (all *p_FDR* ≥ .13).

Among parental caregivers of children with care needs (*n* = 62), 40% reported a mild to moderate care burden while 35% reported no to mild burden, and 24% experienced a high care burden. Care-related quality of life showed a mean score of 9.4, indicating a moderate level of overall well-being in the caregiving role.

### Factors associated with caregiver burden

To examine factors associated with caregiver burden among parental caregivers of children with rare diseases and specific care needs (*n* = 62), three hierarchical multiple linear regression models were computed with caregiver burden as dependant variable (see Table 2).

Model 1 included all six theoretically derived predictors. In this full model, socioeconomic status and care-related quality of life emerged as significant predictors, whereas physical health and unmet supportive care needs showed no meaningful contribution.

Model 2 removed unmet supportive care needs, the predictor with the weakest contribution. As shown in Table 2, model fit remained virtually unchanged, and physical health continued to show no unique explanatory value.

**Table 2:** Multiple linear regression models predicting caregiver burden (ZBI)

**Predictor**	**Model 1 (β)**			**Model 2 (β)**			**Model 3 (β)**		
	*β*	95% CI	*p*	*β*	95% CI	*p*	*β*	95% CI	*p*
Socioeconomic status (Winkler Index)	.359	[.466; 1.207]	<.001	.360	[.473; 1.206]	<.001	.349	[.449; 1.178]	<.001
Depressive symptoms (HADS-D)	.199	[-.035; .856]	.070	.202	[-.024; .856]	.063	.245	[.094; .918]	.017
Care-related quality of life (CarerQol)	-.394	[-2.091;-.504]	.002	-.409	[-2.044; -.655]	<.001	-.457	[-2.146; -.866]	<.001
Treatment satisfaction (ZUF-8)	-.166	[-.825; .022]	.062	-.170	[-.826; .005]	.053	-.197	[-.876; -.075]	.021
Physical health (WHOQOL-BREF)	-.119	[-.189; .062]	.313	-.128	[-.188; .051]	.255			
*R²* (adj.*R²*)	.686 (.652)			.686 (.658)			.678 (.656)		

Note. *N* = 62. Standardized beta coefficients (*β*) are reported from multiple linear regression analyses. Model 1 includes all predictors; Model 2 excludes unmet supportive care needs; Model 3 includes only the strongest predictors from prior models. HADS-D = Hospital Anxiety and Depression ScaleDepression subscale; CarerQol = Care-related quality of life; ZUF-8 = Satisfaction with medical care; WHOQOL-BREF = World Health Organization Quality of Life–Physical Health subscale; SCNS-ST9 = Supportive Care Needs Survey Short Form

Model 3, the final reduced model, excluded physical health and retained the four predictors that contributed meaningfully to explained variance: (1) care-related quality of life (CarerQol), (2) socioeconomic status (Winkler’s Index), (3) depressive symptoms (HADS-D), and (4) satisfaction with medical care (ZUF-8). This model explained a substantial proportion of variance in caregiver burden and has a high goodness-of-fit according to Cohen’s benchmarks for effect size interpretation [27] with *R²* = .68 (*adjusted R²* = .66) and *F*(4, 57) = 30.07 (*p* < .001). Unique contributions of each factor were calculated using semi-partial squared correlations. As shown in Table 2, care-related quality of life accounted for the largest unique share of explained variance (28.0%, *p* = .001), followed closely by socioeconomic status (25.9%, *p* = < .001). Depressive symptoms and treatment satisfaction also made statistically significant contributions, explaining 9.6% and 9.1% of the variance, respectively (*p* = .017 and *p* = .021). The shared variance (-4.8%) reflects overlap between predictors.

## Discussion

### Descriptive analyses and comparison of parental caregivers of children with and without care needs

The descriptive findings highlight a substantial psychosocial burden among parental caregivers. In total, 12% of caregivers showed borderline depressive symptoms and 11% reported clinically relevant levels, with 40% having sought psychological or psychotherapeutic support—patterns consistent with previous work documenting elevated depressive symptoms and distress among parents of children with chronic and rare conditions [28, 29]. Similarly, the high prevalence of unmet supportive care needs observed in both caregiver groups (76.8% reporting at least one unmet need and 37.5% reporting five or more) aligns with previous research documenting substantial informational, emotional, and practical support gaps among parental caregivers of children with chronic or complex health conditions [30, 31]. The domain-specific distribution further indicated that physical, psychological, support-related, and information-related needs were all frequently reported, suggesting broadly elevated and multidimensional caregiving demands in this population.

Among parents of children with care needs, the distribution of caregiver burden—40% reporting mild to moderate burden and 24% reporting high burden—together with moderate care-related quality of life, aligns with research showing similar levels of caregiver burden and reduced well-being among parental caregivers [32]. Collectively, these results underscore the broad psychosocial challenges faced by parental caregivers, even before considering group differences.

Further, exploratory categorization of parent-reported disease-related complaints of children indicated multiple co-occurring functional challenges, including feeding/gastrointestinal, respiratory, musculoskeletal, and neurodevelopmental problems, with cardiovascular/ophthalmologic and multiorgan/congenital manifestations occurring less frequently, highlighting the medically complex care needs faced by many families in this sample. Previous studies in medically complex rare conditions such as oesophageal atresia and cystic fibrosis have reported elevated caregiver burden, higher levels of depressive and anxiety symptoms, and reduced quality of life among parents and caregivers [33–35]. Given that most children in our sample were affected by conditions typically requiring ongoing medical follow-up and repeated surgical interventions, the observed burden may partly be related to medical complexity rather than disease rarity alone. Consequently, the generalisability of our findings to less intervention-intensive rare disease populations may be limited.

In group comparisons, parental caregivers of children with care needs showed trends toward higher depressive symptoms, lower satisfaction with medical care, and moderately increased unmet supportive care needs, although these differences did not reach statistical significance after controlling for multiple comparisons using the false discovery rate (FDR). However, effect sizes for depressive symptoms and treatment satisfaction were in the medium range (*d* = .43–.46), suggesting potentially meaningful differences within this sample despite the lack of statistical significance.

Further, children with rare conditions with care needs were significantly younger, exhibited earlier symptom onset and tended to have higher symptom severity. While younger age may contribute to higher perceived caregiving demands, the observed differences are unlikely to be fully explained by age alone. The earlier symptom onset and greater clinical complexity in this group point to more challenging and resource-intensive caregiving contexts, which may contribute to increased support needs and place greater emotional and logistical burden on caregivers. These results are consistent with systematic reviews on psychosocial impacts in rare disease contexts indicating, that parental caregivers frequently struggle with the unique challenges of navigating diagnostic uncertainty, service coordination and limited healthcare provider expertise, leading to dissatisfaction and unmet health needs and potentially contributing to higher emotional strain and caregiver burden [2, 8, 9, 11]. Further, parental caregivers of children with complex or care-intensive diseases often face substantial financial stress due to medical costs, employment disruptions, and systemic barriers, all of which may negatively affect caregiver quality of life [9, 36].

Overall, these findings support the broader evidence that intensified caregiving demands are associated with elevated psychological burden and reduced quality of life among parental caregivers [7]. Although statistical significance was not observed after FDR adjustment, medium effect sizes suggest potential meaningful differences that warrant further investigation in larger and more diverse samples.

### Factors associated with caregiver burden

Care-related quality of life, socioeconomic status (SES), depression and treatment satisfaction explained a substantial proportion of 68% of the variance in caregiver burden. Care-related quality of life as associated factor showed the strongest effect on caregiver burden, explaining 28% of the variance, comparable to other studies that have found a similarly high association [37]. The central role of care-related quality of life in predicting burden aligns with previous work emphasizing the multidimensional challenges of caregiving for medically complex children [8, 11]. The association remained significant and substantial even when controlling for the other variables, indicating that care-related quality of life plays an independent and meaningful role in explaining caregiver burden, which is in line with other findings [38]. However, we assume that this high explanatory power could also be attributed to the fact that quality of life encompasses central aspects of caregiving, so that a certain degree of conceptual overlap between these constructs seems plausible and has already been addressed in other studies [39, 40]. It is therefore conceivable that future predictor analyses may benefit from a more distinct theoretical delineation of these constructs.

Interestingly, and in contrast to findings from general caregiving studies, higher SES was associated with greater caregiver burden in our sample. While low SES is typically linked to structural disadvantages and poorer health outcomes [5, 41], caregivers with higher SES may experience increased role conflict due to simultaneous professional and caregiving responsibilities, particularly in demanding occupations [42, 43]. This “double burden” could heighten stress despite financial or structural advantages [44, 45]. However, as our sample predominantly consisted of parents with medium to high SES, this pattern should be interpreted with caution. Lower SES groups with relevant caregiving burdens may be underrepresented, potentially limiting the generalizability of these findings beyond the present sample.

The finding that depressive symptoms significantly predicted burden is well supported by previous work, highlighting the reciprocal relationship between psychological distress and caregiving demands [8, 46]. Depression not only reflects emotional strain but may also compromise caregivers’ coping capacity, exacerbate perceived burden, and reduce access to social support.

Lower satisfaction with care was also significantly associated with perceived burden, echoing recent evidence that caregivers of children with rare diseases often encounter fragmented care pathways, lack of coordination, and inadequate communication with providers [9]. Enhancing health care responsiveness and implementing rare disease care navigators may alleviate this dissatisfaction and, in turn, reduce caregiver burden.

Depressive symptoms and treatment satisfaction reached significance only after model reduction, reflecting greater precision of estimates once non-contributing variables were removed, rather than instability or absence of their effects. Physical health and unmet supportive care needs were excluded because they did not explain unique variance in caregiver burden beyond the remaining variables. This likely reflects that physical health and unmet supportive care needs shared variance with other variables—particularly depressive symptoms and care-related quality of life—and showed limited variability, which may have reduced the visibility of their associations with caregiver burden in cross-sectional analyses but could become more apparent in longitudinal or mediation-focused studies.

Overall, care-related quality of life and socioeconomic status accounted for the largest portion of variance in caregiver burden, with depressive symptoms and treatment satisfaction contributing more modestly, indicating that future studies may benefit from examining both primary and secondary determinants to better understand caregiver burden.

### Limitations

This study has several limitations that should be considered when interpreting the findings. First, the use of convenience sampling through centres with a favourable pre-assessment of their intersectoral management concept likely resulted in a sample biased toward families receiving relatively high levels of specialized healthcare. As a result, the reported levels of depressive symptoms and caregiver burden may be lower than those experienced by caregivers without access to comparable resources.

Second, the sample composition may limit generalizability. Participants were predominantly of German citizenship (98.4%) and from medium to high socioeconomic backgrounds. In addition, the study included families affected by a limited range of rare conditions and was conducted exclusively in Germany. Given potential differences in healthcare systems, diagnostic pathways, and support structures across countries and disease groups, the findings should be generalized to other rare disease contexts with caution. Nevertheless, the observed sample characteristics are consistent with those reported in similar studies in the field [29, 47].

Furthermore, the sample predominantly included children with medically complex rare conditions characterised by repeated medical or surgical interventions (e.g., cystic fibrosis, esophageal atresia, VACTERL association). This may further limit comparability with studies focusing on less intervention-intensive rare disease populations and should be considered when interpreting the findings. The relatively small and uneven distribution of individual diagnostic groups further limited the ability to conduct meaningful disease-specific subgroup analyses. Moreover, symptom clusters were derived from brief parent-reported free-text responses and did not involve standardized assessments of functional impairment, cognitive functioning, school participation, or disease severity. Therefore, these findings should be interpreted as exploratory indicators of care-related complexity rather than validated clinical classifications.

The vast majority (84%) of respondents were mothers, reflecting persistent gendered caregiving norms, where women disproportionately assume caregiving responsibilities [5, 48]. While this is consistent with other caregiver research in rare diseases it limits conclusions regarding the experiences of fathers as primary caregivers, which may differ [49].

Thirdly, the cross-sectional design of the study precludes causal inferences. Information on the time elapsed between diagnosis and study participation was not available, preventing examination of whether parental depressive symptoms were related to more recent diagnoses or changes in distress over the diagnostic trajectory. Depressive symptoms may therefore have preceded study participation. While regression-based imputation was used to address missing data under the assumption of data missing at random, this assumption cannot be verified. The modest sample size also poses limitations for generalizability, although this constraint is common in rare disease research due to the inherently small population sizes. Future studies should consider employing longitudinal designs and mixed method approaches to gain a more nuanced understanding of caregiver burden trajectories and the pathways through which socioeconomic status affects caregiving experiences in rare disease contexts, while also including a broader range of rare conditions and investigate caregivers across different countries and healthcare systems to improve the generalizability of findings.

## Conclusion

This study provides important insights into the caregiver burden experienced by parental caregivers of a child with a rare disease within a heterogeneous but limited range of conditions represented in this sample. Overall, a substantial proportion of parents reported depressive symptoms and unmet supportive care needs, and a notable subgroup experienced high caregiver burden. Children with rare conditions with care needs were significantly younger, exhibited earlier symptom onset and tended to have higher symptom severity. Lower care-related quality of life and higher socioeconomic status emerged as the strongest factors associated with caregiver burden, alongside parental depressive symptoms and treatment satisfaction.

These findings in this sample are not fully consistent with traditional assumptions that caregiver burden is primarily linked to low-resource contexts and highlight the complex, multidimensional nature of caregiving in rare disease contexts as represented here. They underscore the need for holistic, family-centred interventions targeting the factors most strongly associated with burden. The observed association between higher socioeconomic status with greater burden suggests that caregiver burden may not be restricted to traditionally assumed high-risk groups; however, this pattern should be interpreted cautiously given the underrepresentation of lower SES families in this sample. Future studies should validate these findings in larger, more diverse, and longitudinal samples across different healthcare systems.

## Data Availability

Data are available from the authors upon reasonable request and after consultation of the data protection manager of the University Medical Center.
